# Primary-Tumor Transcriptomic Signatures Associated with Circulating Tumor Cells Status in Breast Cancer: From CTC-Negative Remodeling to CTC-Associated Metastatic Programs

**DOI:** 10.3390/biomedicines14102246

**Published:** 2026-10-04

**Authors:** Betul Budak, Sena Ozay, Betul Yilmaz, Kazim Yalcin Arga

**Affiliations:** 1 Department of Bioengineering, Faculty of Engineering, Marmara University, Istanbul 34854, Türkiye; betul.budak@marmara.edu.tr; 2 Department of Bioengineering, Faculty of Life and Natural Sciences, Abdullah Gül University, Kayseri 38080, Türkiye; senaozaay@gmail.com; 3 Department of Medical Biochemistry, Faculty of Medicine, Marmara University, Istanbul 34854, Türkiye; 4 Genetic and Metabolic Diseases Research and Investigation Center, Marmara University, Istanbul 34854, Türkiye; 5 Department of Biochemistry, School of Medicine, Recep Tayyip Erdogan University, Rize 53100, Türkiye; 6 Center for Nanotechnology & Biomaterials Applications and Research, Marmara University, Istanbul 34854, Türkiye

**Keywords:** breast cancer, circulating tumor cells, drug repurposing, immune evasion, metastasis, transcriptomics

## Abstract

**Background/Objectives**: Circulating tumor cells (CTCs) play a central role in metastasis by enabling tumor dissemination through the bloodstream. However, the molecular transition from CTC-negative (CTC^−^) to CTC-positive (CTC^+^) states and their associated systemic remodeling remain poorly understood. **Methods**: We performed a transcriptomic comparison between CTC^+^, CTC^−^, and healthy individuals using RNA-seq data and a bioinformatics pipeline. Differentially expressed genes (DEGs) were subjected to functional enrichment, protein–protein interaction (PPI) analysis, survival modeling, and drug perturbation prediction to delineate the molecular determinants of CTC emergence and persistence. **Results**: Primary tumors from CTC^+^ patients exhibited distinct transcriptomic profiles characterized by the upregulation of genes involved in PI3K-Akt signaling, extracellular matrix organization, and chromatin regulation (e.g., *HDAC1*, *JUN*, *KLK10*), alongside the downregulation of immune activation pathways, including complement, B-cell receptor (BCR), and T-cell receptor (TCR) signaling. Multi-gene survival models derived from upregulated (*KLK10*, *MEOX2*, *IRS4*, *RND2*) and downregulated (*ALDH3A1*, *PPP2R2B*, *IGF1R*) hub genes significantly stratified patient outcomes (HR = 1.74–2.44, *p* < 0.01). In contrast, primary tumors from CTC^−^ patients displayed early systemic transcriptional alterations relative to healthy controls, with 1700+ DEGs linked to MAPK/ERK and receptor tyrosine kinase (RTK) signaling pathways, suggesting pre-metastatic immune reprogramming even before CTC detection. Drug repurposing analysis identified Trichostatin A, BX-795, and Alvocidib as candidate compounds capable of reversing the CTC^+^ transcriptional signature. **Conclusions**: Rather than supporting a strictly binary view of CTC status, our findings are consistent with a transcriptional continuum of distinct yet partially overlapping primary-tumor transcriptional states associated with CTC^−^ and CTC^+^ status, highlighting a molecular shift from immune suppression to epigenetic adaptation that facilitates hematogenous dissemination. The identified multi-gene prognostic panels and small-molecule candidates provide a foundation for CTC-targeted therapeutic strategies and early detection biomarkers in breast cancer.

## 1. Introduction

Metastasis remains the leading cause of cancer-related mortality, accounting for nearly 90% of deaths among breast cancer patients [1,2]. Central to this process are circulating tumor cells (CTCs), rare tumor-derived cells that detach from the primary tumor, enter the peripheral bloodstream, and eventually seed distant organs [3]. Despite their low abundance, CTCs are considered powerful biomarkers of disease progression, therapeutic resistance, and relapse [4]. However, the molecular evolution that enables tumor cells to transition from a non-circulating (CTC^−^) state to a fully competent, circulation-surviving (CTC^+^) phenotype remains incompletely understood.

Previous studies have highlighted that CTCs display substantial phenotypic and molecular heterogeneity, encompassing epithelial, mesenchymal, and hybrid cell states that facilitate plasticity and immune escape [5,6]. Current CTC research has therefore increasingly shifted from simple enumeration toward molecular characterization, with transcriptomic profiling providing insights into cellular plasticity, immune evasion, and metastasis-associated adaptation. Nonetheless, the transcriptional reprogramming associated with CTC-negative and CTC-positive states, particularly across the continuum from CTC^−^ to CTC^+^, has not been systematically characterized. Understanding this progression is crucial for identifying early biomarkers and therapeutic targets capable of intercepting metastatic dissemination.

CTC^+^ cells face a uniquely hostile environment within the bloodstream, encountering oxidative stress, immune surveillance, and shear forces. To survive, they must activate adaptive pathways involving cytoskeletal remodeling, metabolic reprogramming, and immune evasion [7]. In contrast, CTC^−^ patients, who lack detectable circulating cells, may already exhibit systemic molecular alterations reflecting tumor–host interactions or pre-metastatic niche formation. Indeed, recent transcriptomic studies suggest that such changes in peripheral blood cells could precede the physical detection of CTCs, indicating a potential “pre-CTC” molecular phase of metastasis initiation [8,9,10]. However, to our knowledge, no previous study has systematically integrated transcriptomic comparisons of CTC^−^, CTC^+^, and healthy groups within a common analytical framework to determine whether these states are associated with a coordinated continuum of immune, signaling, and epigenetic remodeling.

In this study, we sought to elucidate the molecular continuum from CTC^−^ to CTC^+^ states by integrating transcriptomic data from breast cancer patients and healthy individuals. Using high-throughput RNA-seq datasets, we performed differential gene expression and systems-level analyses, incorporating pathway enrichment, protein–protein interaction (PPI) network construction, survival modeling, and drug perturbation prediction. Our objective was to (1) identify the key biological processes and hub genes that distinguish CTC^+^ from CTC^−^ phenotypes, (2) characterize systemic reprogramming events in CTC^−^ patients relative to healthy controls, and (3) propose candidate therapeutic compounds that could reverse the CTC^+^ molecular signature. The results reveal that CTC^+^ cells exhibit profound immune suppression, chromatin remodeling, and signaling activation (e.g., PI3K-Akt, ECM–CAM interactions), while CTC^−^ patients already display pre-metastatic transcriptional reprogramming involving MAPK and RTK pathways. Together, these findings support a working model in which CTC-associated molecular states form a transcriptional continuum rather than a strictly binary transition.

## 2. Materials and Methods

### 2.1. Data Acquisition and Processing

RNA sequencing (RNA-seq) data were retrieved from the NCBI Sequence Read Archive (SRA) database [BioProject PRJNA839244] [11]. The RNA-seq profiles were generated from fresh-frozen primary breast tumor tissues, together with normal breast tissue controls. The CTC status was determined preoperatively based on the detection of CTCs with epithelial-to-mesenchymal transition characteristics (CTC_EMT) using quantitative RT-PCR for the EMT-associated genes *TWIST1, SNAIL1, SLUG*, and *ZEB1*.

Samples included in the present analysis were categorized into three groups according to the reported CTC status: (i) CTC-positive (CTC^+^, *n* = 8), (ii) CTC-negative (CTC^−^, *n* = 9), and (iii) normal breast tissue (healthy) controls (*n* = 5). The molecular characteristics of the analyzed cohort according to CTC status are summarized in Table 1. Given the limited number of samples within individual molecular subtypes, these variables (i.e., hormone receptor status, HER2 status, Ki-67 status, and molecular subtype) were used primarily for descriptive characterization and were not incorporated as covariates into the differential-expression models.

Raw .sra files were converted to .fastq with the SRA Toolkit (v3.2.1) [12], aligned to GRCh38 with HISAT2 (v2.2.1) [13], and converted/sorted to BAM with Samtools (v1.17) [14]. Gene-level read counts were generated using featureCounts from the Rsubread package (v2.20.0) [15] with the *Homo sapiens* GTF (Ensembl release 110) [16]. Count matrices were consolidated for downstream statistical analysis in R (v4.3.1).

### 2.2. Differential Gene Expression Analysis

Differential expression analysis was performed using the DESeq2 package (v1.46.0) [17]. Raw count data were processed with DESeq2’s default workflow, which models read counts with a negative binomial distribution and applies normalization using the median-of-ratios method to account for library size differences. Samples were categorized into three experimental groups (CTC^+^, CTC^−^, and Healthy). Pairwise comparisons were made between CTC^+^ vs. CTC^−^, and CTC^−^ vs. normal control (healthy) groups. Genes with a Benjamini–Hochberg-adjusted p-value (padj) < 0.05 and |log_2_ fold change| > 1 were considered significantly differentially expressed. Results were visualized using volcano plots, heatmaps, and t-distributed stochastic neighbor embedding (t-SNE) clustering. Variance-stabilizing transformation (VST) was applied to the count matrix prior to dimensionality reduction and visualization.

### 2.3. Functional Enrichment Analysis

Significant upregulated and downregulated genes were subjected to functional enrichment analysis using the ConsensusPathDB platform [18]. Both Gene Ontology (GO) categories (biological process, molecular function, cellular component) [19] and pathway databases, KEGG [20] and Reactome [21], were interrogated. The enrichment results were ranked according to statistical significance and enrichment score. To account for multiple testing across functional categories, false discovery rate (FDR)-adjusted values were used, and terms with an adjusted *p*-value (q-value) < 0.05 were considered significantly enriched.

### 2.4. Protein–Protein Interaction (PPI) Network and Hub Gene Identification

To explore molecular interaction architecture, PPI networks were constructed using background data from STRING [22], BioGRID [23], and IntAct [24]. Filtered DEG lists were imported into Cytoscape (v3.10.3) [25], where CytoHubba (v0.1) [26] was used to identify hub genes based on topological metrics including degree and betweenness connectivity. Biologically relevant modules were further extracted using the MCODE plugin (v2.0.3) [27], and the most interconnected clusters were visualized for interpretation. This step enabled the identification of CTC^+^-specific and CTC^−^-specific regulatory cores, shedding light on their distinct functional signatures.

### 2.5. Survival Analysis and Prognostic Model Construction

To evaluate prognostic relevance, Kaplan–Meier survival analyses were performed using SurvExpress [28] with the TCGA-BRCA (Breast Invasive Carcinoma, July 2016 release) dataset obtained from The Cancer Genome Atlas: https://www.cancer.gov/tcga (accessed on 9 August 2025). Candidate hub genes were used to construct multi-gene prognostic models. For each gene panel, SurvExpress generated a prognostic index (PI) using a Cox proportional hazards model, in which gene-expression values were weighted by their corresponding Cox regression coefficients. The resulting PI therefore represents the linear combination of the expression values of the genes included in the model. Patients were ordered according to their PI and dichotomized at the median PI into low-risk and high-risk groups. Survival differences were evaluated using log-rank tests, and hazard ratios (HRs) and concordance indices (C-indices) were calculated to assess prognostic performance.

### 2.6. Small-Molecule Drug Prediction

Potential compounds capable of reversing the CTC^+^ and CTC^−^ gene expression profiles were identified using the L1000CDS^2^ platform [29]. This approach compares the directionality of disease-associated gene signatures with drug-induced transcriptional responses from the LINCS database. Compounds with the highest reversal scores were shortlisted as candidates for further investigation. The sensitivities of BRCA cell lines to the candidate compounds were then evaluated using IC_50_ (μM) values obtained from the Genomics of Drug Sensitivity in Cancer (GDSC) database [30]. Based on quartile distribution, cell lines within the lowest 25% of IC_50_ values were classified as sensitive, those within the 25–75% range as intermediate, and those within the highest 25% as resistant. The present analysis was restricted to small-molecule perturbagens represented in the L1000CDS^2^/LINCS framework and did not systematically evaluate antibody- or peptide-based therapeutic agents.

### 2.7. Data Visualization and Statistical Analysis

All data processing, normalization, and visualization were performed in RStudio (v2023.09) using the following packages: DESeq2 (v1.46.0), ggplot2 (v3.5.2), pheatmap (v1.0.13), Rtsne (v0.17), and clusterProfiler (v4.14.6). Statistical significance was determined at adjusted *p*-value < 0.05 unless otherwise stated.

## 3. Results

### 3.1. CTC^+^ and CTC^−^ Samples Exhibit Distinct Global Transcriptomic Profiles

**Figure 1:**
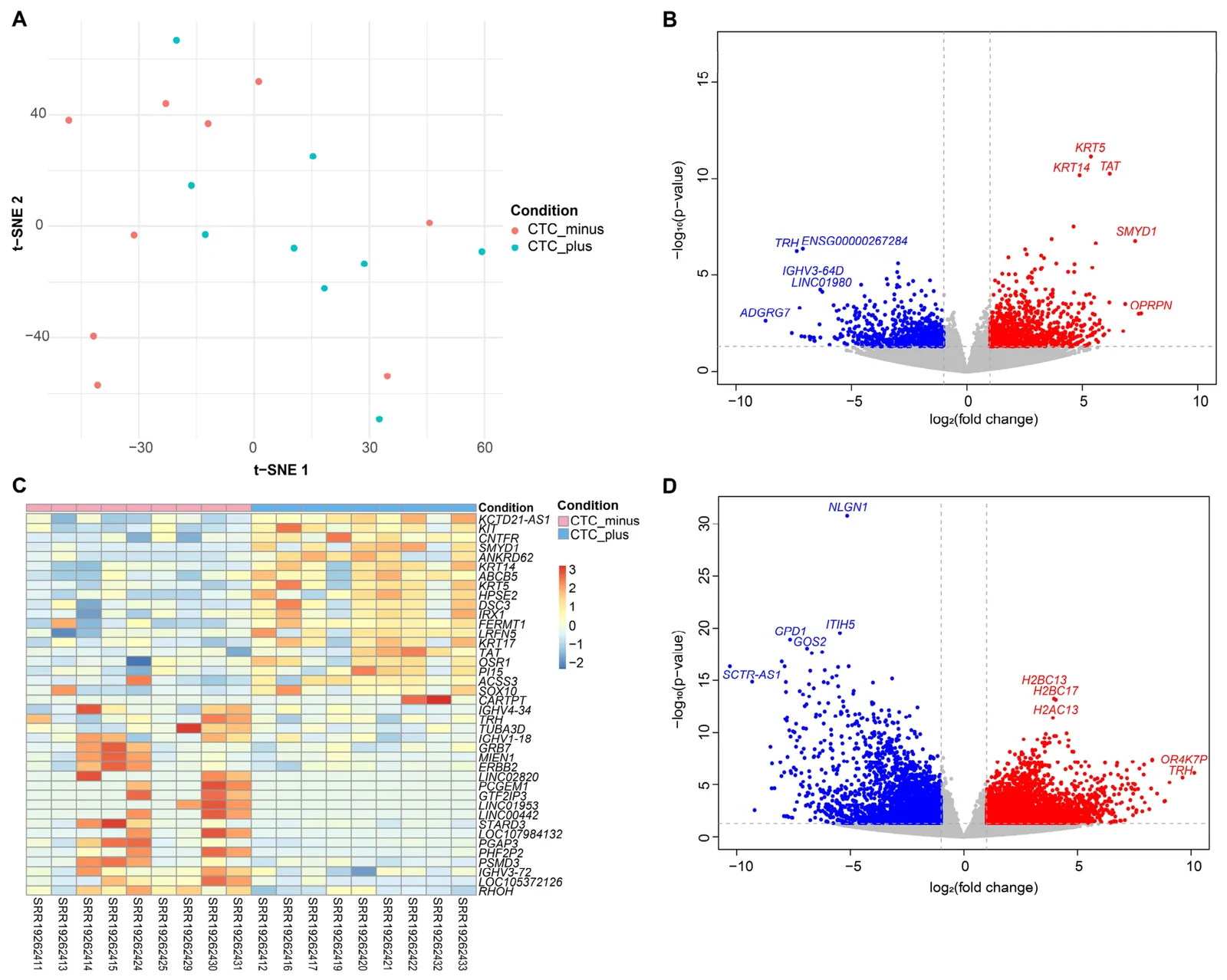
Transcriptomic profiling of primary breast tumors according to circulating tumor cell (CTC) status. (**A**) T-distributed stochastic neighbor embedding (t-SNE) plot showing global gene expression patterns of primary tumor samples from CTC^+^ (blue dots) and CTC^−^ (red dots). (**B**) Volcano plot of differentially expressed genes in primary tumors from CTC^+^ versus CTC^-^ samples. (**C**) Heatmap showing hierarchical clustering of CTC^+^ and CTC^-^ breast cancer samples according to the expression levels of the top 40 most significantly altered genes. (**D**) Volcano plot of differentially expressed genes in primary tumors from CTC^−^ samples versus normal breast tissue controls. Differentially expressed genes were identified using adjusted *p*-value < 0.05 and |log_2_(fold change)| > 1. Genes with log_2_(fold change) > 1 were considered upregulated and are represented in red, whereas genes with log_2_(fold change) < −1 were considered downregulated and are represented in blue. Genes that did not meet both criteria are shown in grey.

Global transcriptomic profiling revealed meaningful differences between circulating tumor cell–positive (CTC^+^) and circulating tumor cell–negative (CTC^−^) breast cancer samples. Dimensionality reduction using t-distributed stochastic neighbor embedding (t-SNE) demonstrated a clear overall tendency for the two groups to separate into distinct regions of the embedding space (Figure 1A). However, a small number of samples were positioned closer to the opposite group, indicating that although CTC status strongly influences transcriptomic patterns, the separation between CTC^+^ and CTC^−^ states is not completely discrete. This partial overlap suggests the presence of intra-group biological heterogeneity as well as transitional transcriptional states that may reflect early or intermediate remodeling events.

Despite this limited intermixing, the dominant clustering pattern supports the notion that CTC^+^ samples undergo substantial transcriptomic reprogramming distinct from CTC^−^ samples. The spatial distribution observed in the t-SNE plot is consistent with the concept of a transcriptional continuum, in which the CTC^−^ and CTC^+^ groups represent distinct yet partially overlapping molecular states rather than strictly discrete phenotypes. This global segregation provided the basis for subsequent differential expression, functional enrichment, and network-based analyses aimed at resolving the molecular determinants of CTC emergence and persistence.

### 3.2. Differential Gene Expression Reveals Major Transcriptional Reprogramming Associated with CTC^+^ Status

Subsequent differential gene expression analysis using the DESeq2 package identified extensive transcriptional changes between CTC^+^ and CTC^−^ conditions. In total, 704 genes were significantly upregulated and 480 genes were significantly downregulated in the CTC^+^ samples, using a threshold of adjusted *p* < 0.05 and |log_2_FC| > 1. The resulting volcano plots (Figure 1B) demonstrated the scale and statistical robustness of these alterations, highlighting clusters of genes that exhibit either marked overexpression or suppression in the presence of CTCs. To visualize how these differences contribute to sample clustering, a heatmap of the top 40 most significantly altered genes was generated (Figure 1C). The heatmap revealed a high degree of internal coherence, with CTC^+^ and CTC^−^ samples forming two distinct clusters based on their expression signatures. Among the most prominent upregulated genes were *HDAC1*, *JUN*, *APP*, and *ACTN2*, all of which are known to participate in processes related to chromatin remodeling, cytoskeletal dynamics, and transcriptional regulation. Conversely, the downregulated subset included *ALDH3A1*, *PPP2R2B*, *CD79A*, and *IGHG1*, which are associated with immune response and tumor-suppressive signaling. These coordinated differences further support the presence of distinct molecular states associated with CTC status within the proposed continuum framework.

### 3.3. Functional Enrichment Indicates Activation of Oncogenic Pathways and Immune Suppression in Primary Tumors from CTC^+^ Patients

**Figure 2:**
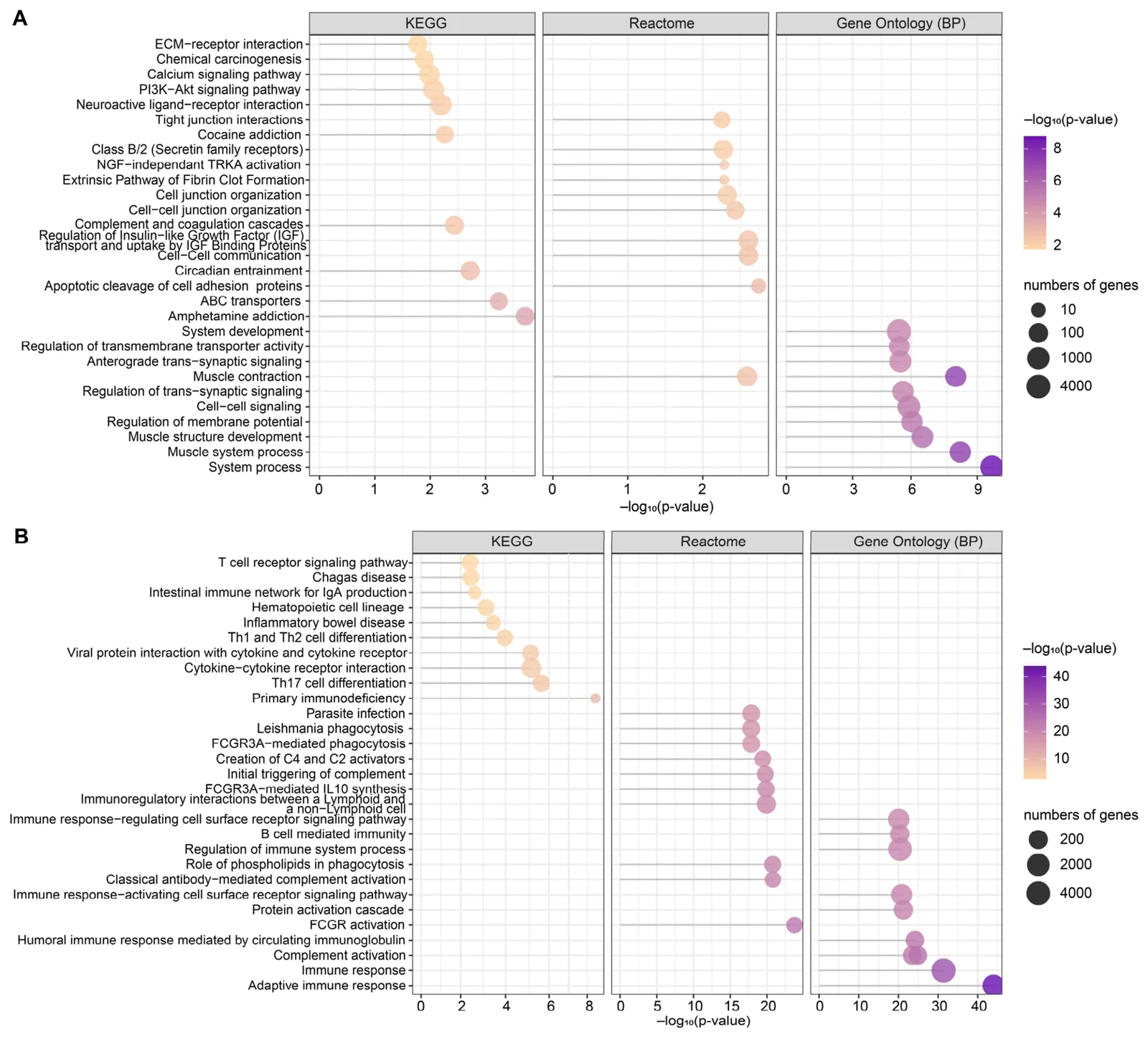
Functional enrichment analysis of differentially expressed genes primary tumors according to CTC status. (**A**) Significantly enriched KEGG, Reactome, and Gene Ontology Biological Process (GO-BP) terms among genes upregulated in primary tumors from CTC^+^ patients relative to CTC^−^ patients. (**B**) Significantly enriched KEGG, Reactome, and GO-BP terms among genes downregulated in primary tumors from CTC^+^ patients relative to CTC^−^ patients. The x-axis and bubble color represent −log_10_(*p*-value), with higher values indicating greater statistical significance, while bubble size represents the number of genes associated with each enriched term.

Functional enrichment analysis further delineated the biological implications of these transcriptional shifts. Upregulated genes in CTC^+^ samples were predominantly enriched in pathways linked to PI3K-Akt signaling, extracellular matrix (ECM) organization, cell adhesion molecules (CAMs), ABC transporters, and neuroactive ligand–receptor interactions (Figure 2A). These pathways collectively contribute to enhanced cellular adaptability, motility, and survival within the bloodstream. The activation of PI3K-Akt signaling and ECM remodeling pathways supports the hypothesis that CTCs require not only mechanical resilience but also dynamic remodeling of adhesion and signaling machinery to withstand circulatory stress and immune surveillance. The enrichment of neuroactive and synaptic-like signaling processes, though unexpected, suggests that CTCs may adopt a neuron-like transcriptional phenotype to facilitate rapid responsiveness to external cues, consistent with emerging evidence of neural mimicry in metastatic cells.

In contrast, downregulated genes were enriched in immune and inflammatory pathways, including complement activation, antigen processing and presentation, cytokine–cytokine receptor interactions, and B-cell receptor (BCR) and T-cell receptor (TCR) signaling cascades (Figure 2B). This coordinated silencing of immune-related pathways indicates a transcriptionally mediated immune evasion strategy. Genes involved in adaptive immunity, such as *IGHG1*, *IGHG3*, *CD79A*, and *PPP2R2B*, were among the most significantly repressed, suggesting that primary tumors associated with CTC^+^ status exhibit reduced expression of molecular features involved in adaptive immune recognition. The suppression of complement and phagocytosis-related genes further reinforces the concept that CTCs minimize immune visibility during hematogenous dissemination. Together, these pathway-level changes define an immune-to-adaptive molecular shift that is consistent with the proposed CTC-associated continuum.

### 3.4. Protein–Protein Interaction (PPI) Networks Identify Distinct Regulatory Modules Associated with CTC^+^ Status

**Figure 3:**
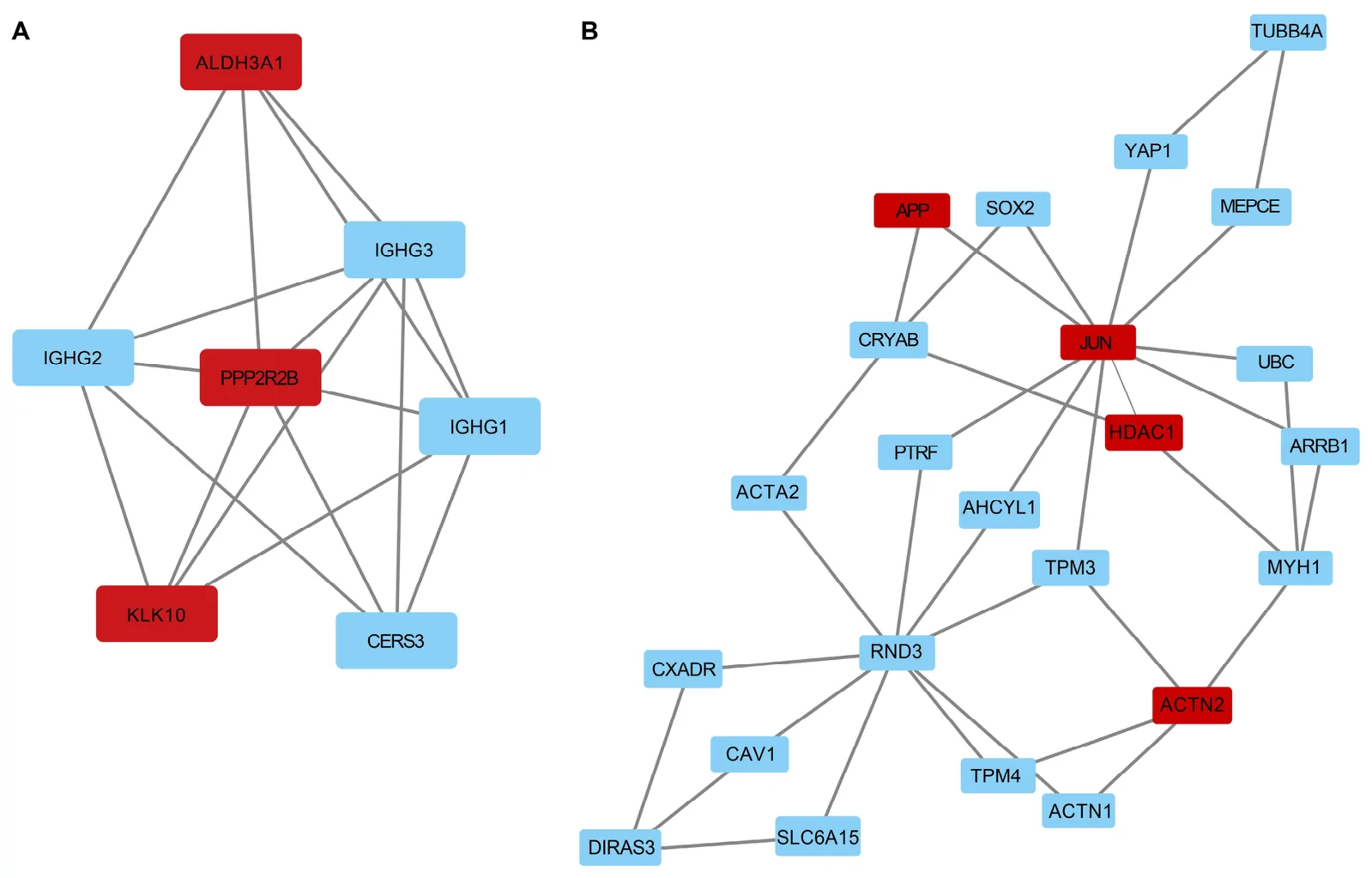
Protein–protein interaction subnetworks in CTC^+^ samples. (**A**) Downregulated gene module. (**B**) Upregulated gene module.

To explore the regulatory structure underlying these expression patterns, protein–protein interaction (PPI) networks were constructed using STRING and visualized in Cytoscape. Distinct subnetworks emerged for upregulated and downregulated gene sets (Figure 3). The downregulated module featured genes such as ALDH3A1, PPP2R2B, and KLK10, interconnected through immune response and oxidative stress detoxification pathways. The observed dense interconnectivity suggested that these genes operate as part of a coordinated immune-regulatory program that is transcriptionally repressed in CTC^+^ cells. On the other hand, the upregulated network, centered on HDAC1, JUN, APP, and ACTN2, displayed high modularity and was enriched in pathways related to transcriptional activation, cytoskeletal rearrangement, and chromatin regulation. The central positioning of HDAC1 within this module points to a potential epigenetic master regulator driving CTC plasticity and adaptive survival. This observation aligns with the increasing recognition of chromatin remodeling enzymes as determinants of metastasis competence. At the network level, the contrast between immune-associated repression and epigenetic/transcriptional activation further supports the continuum framework emerging from the differential-expression and enrichment analyses.

### 3.5. Prognostic Gene Signatures Associated with CTC^+^ Status Stratify Breast Cancer Patients

**Figure 4:**
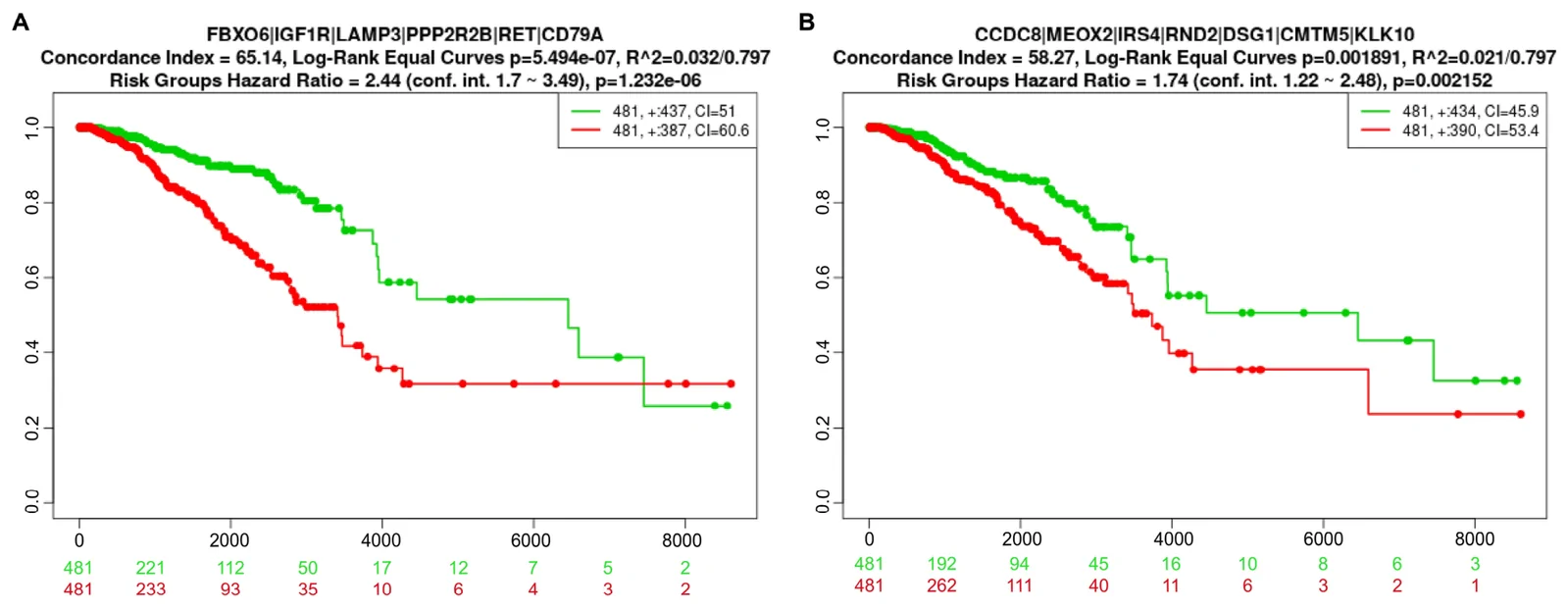
Prognostic stratification based on CTC^+^ gene signatures. (**A**) Kaplan–Meier survival curves constructed from the expression of downregulated hub genes (*FBXO6*, *IGF1R*, *LAMP3*, *PPP2R2B*, *RET*, and *CD79A*) with a hazard ratio of 2.44 (*p* = 1.23 × 10^−6^). (**B**) Kaplan–Meier survival curves constructed from the expression of upregulated hub genes (*KLK10*, *MEOX2*, *IRS4*, *RND2*, *DSG1*, *CMTM5*, and *CCDC8*) with a hazard ratio of 1.74 (*p* = 0.00215). Patients were divided at the median prognostic index into high-risk (red) and low-risk (green) groups.

Survival analyses using the TCGA-BRCA cohort provided independent evidence of prognostic association for these gene signatures. Kaplan–Meier models constructed from downregulated hub genes, including *FBXO6*, *IGF1R*, *LAMP3*, *PPP2R2B*, *RET*, and *CD79A*, significantly stratified overall survival (hazard ratio [HR] = 2.44, *p* = 1.23 × 10^−6^), with lower expression levels correlating with poorer overall survival (Figure 4A). Similarly, a multigene panel composed of upregulated hub genes (*KLK10*, *MEOX2*, *IRS4*, *RND2*, *DSG1*, *CMTM5*, *CCDC8*) yielded a hazard ratio of 1.74 (*p* = 0.00215) (Figure 4B). Importantly, these multigene combinations demonstrated greater prognostic strength than individual genes alone, underscoring the relevance of network-based biomarker strategies. The gene *ALDH3A1*, which appeared as a downregulated node in the immune-associated cluster, was particularly noteworthy; patients with low *ALDH3A1* expression exhibited a hazard ratio of 6.51, suggesting that its suppression may contribute to aggressive disease behavior, potentially through reduced oxidative stress resistance.

### 3.6. Primary Tumors from CTC^−^ Patients Exhibit Distinct Transcriptomic Remodeling Relative to Normal Breast Tissue

Parallel analysis of primary tumor tissues from CTC^−^ patients versus normal breast tissue controls revealed substantial transcriptomic remodeling even in the absence of detectable circulating tumor cells. Differential expression analysis identified 1701 upregulated and 1738 downregulated genes in CTC^−^ tumors relative to normal controls (Figure 1D). The t-SNE plots indicated a distinct clustering pattern separating these two groups, demonstrating substantial transcriptional differences associated with the CTC^−^ tumor state. Enrichment analysis revealed activation of pathways such as MAPK/ERK signaling, receptor tyrosine kinase (RTK) activity, and stress response networks, while immune and metabolic regulation pathways were partially suppressed. This pattern is consistent with tumor-associated molecular remodeling that may be relevant to subsequent dissemination; however, the cross-sectional design does not establish that these changes temporally precede CTC emergence. In line with these observations, survival modeling showed that both upregulated (*RTKN*, *ACTC1*, *FAM110A*) and downregulated (*PDHA1*, *NUPR1*, *YBX1*, *CCDC8*) gene panels could significantly stratify patient survival outcomes, supporting their potential prognostic relevance. Thus, the CTC^−^ versus normal controls comparison provides an additional molecular state that supports, without establishing a temporal sequence, the proposed continuum framework.

### 3.7. Drug Repurposing Analysis Identifies Candidate Compounds Targeting CTC Signatures

**Figure 5:**
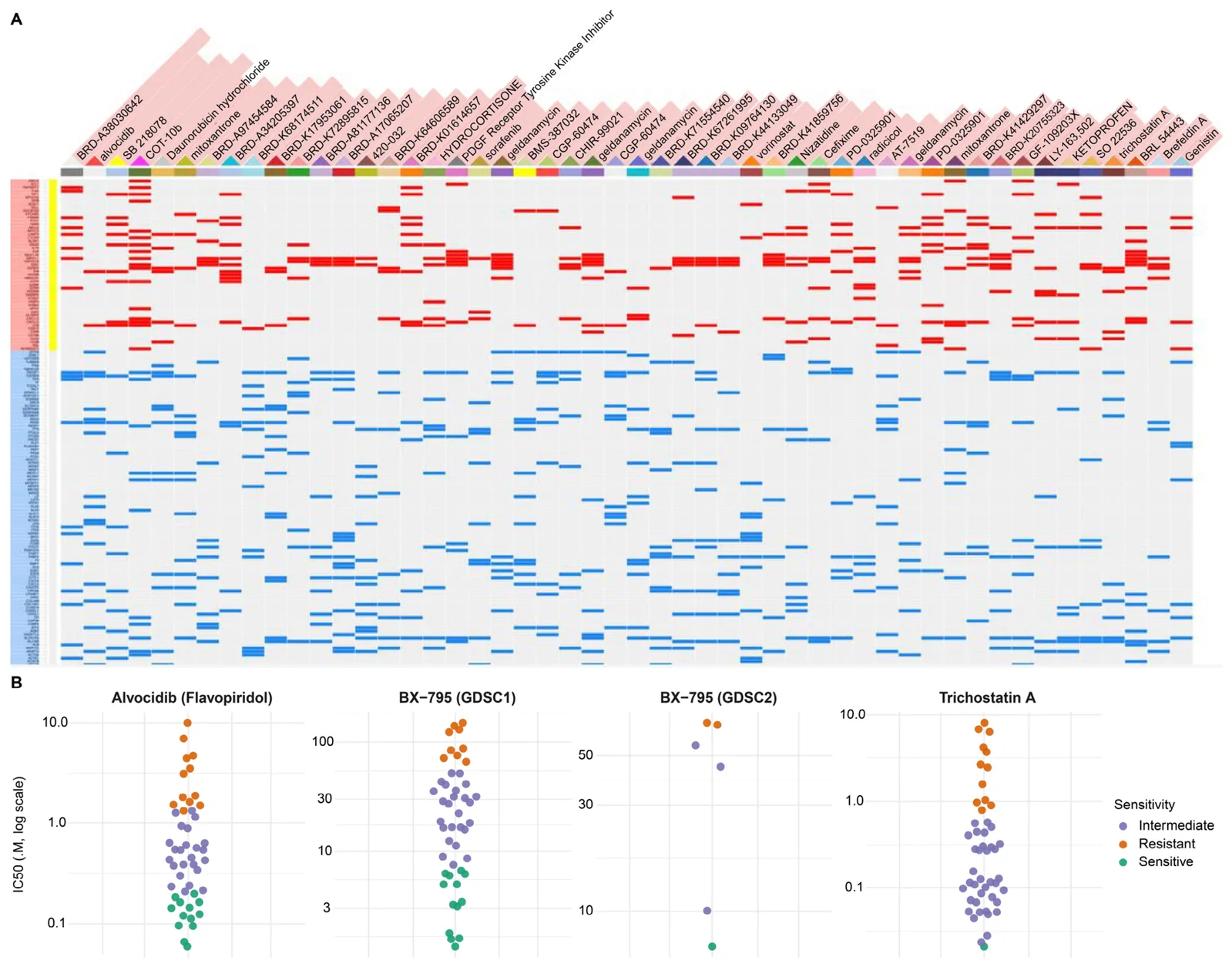
Drug repurposing and sensitivity analysis based on CTC+ gene signatures. (**A**) Top 50 in silico candidate compounds predicted by L1000CDS^2^ to reverse the transcriptional signature of CTC^+^ samples. The input gene signature is displayed as rows of the matrix, with red and blue label bars indicating upregulated and downregulated genes, respectively. (**B**) Sensitivity profiles of BRCA cell lines to the candidate compounds based on IC_50_ (μM) values obtained from the Genomics of Drug Sensitivity in Cancer (GDSC). Cell lines were classified as sensitive (green), intermediate (purple), or resistant (orange).

To identify therapeutic opportunities, the CTC^+^ and CTC^−^ expression signatures were analyzed using the L1000CDS^2^ platform. This approach yielded several compounds with gene expression reversal potential (Figure 5). Among the top-ranked hits for the CTC^+^ signatures were Trichostatin A (a histone deacetylase inhibitor), BX-795 (a TBK1/IKKε inhibitor), and Alvocidib (a cyclin-dependent kinase inhibitor). Each of these compounds targets core pathways implicated in CTC maintenance, such as epigenetic suppression, inflammatory signaling, and proliferative control. Interestingly, Trichostatin A appeared across multiple analytical thresholds, reinforcing the relevance of epigenetic modulation as a therapeutic axis in CTC biology.

The sensitivities of BRCA cell lines to these compounds were evaluated based on their IC50 (μM) values in the GDSC database, revealing that 22 BRCA cell lines with distinct molecular characteristics were sensitive to at least one of the compounds (Table 2). Among the compounds tested, only the MCF7 cell line (IC50 = 0.02 μM) was found to be sensitive to Trichostatin A, while the remaining cell lines were classified as intermediate or resistant. Alvocidib exhibited potent cytotoxic activity across a broad panel of BRCA cell lines, with IC50 values ranging from 0.06 to 0.20 μM, indicating high sensitivity at submicromolar concentrations. In contrast, BX-795 showed IC50 values between 1.3 and 6.9 μM, suggesting that higher concentrations were required to achieve cytotoxic effects.

For the CTC^−^ expression profile, compounds such as Foretinib, PD-184352, and NVP-AUY922 were identified, all targeting MAPK and RTK signaling pathways, consistent with the observation that these pathways are active prior to CTC detection. Collectively, these findings suggest that multi-targeted drug repurposing strategies could be employed to pharmacologically reverse the transcriptional features characteristic of both early and advanced stages of CTC evolution.

### 3.8. Integrative Model of CTC Emergence and Systemic Remodeling

Integrating these findings, a unifying model of CTC biology emerges. The observed cross-sectional transcriptional patterns are consistent with a molecular continuum linking CTC^−^ and CTC^+^ states rather than a strictly discrete shift. In this working model, tumor-associated systemic cues may be associated with a pre-metastatic transcriptional program, manifesting as CTC^−^-specific alterations in signaling and immune regulation. CTC^+^ status is subsequently associated with an additional layer of transcriptional remodeling, marked by immune silencing, epigenetic activation, and enhanced survival signaling. The convergence of this profile with metastatic breast cancer transcriptomes suggests that CTCs are not passive byproducts but active intermediates in metastatic dissemination. These results collectively provide a high-resolution systems-level view of CTC biology and lay the groundwork for biomarker-driven strategies aimed at early detection and targeted intervention in metastatic breast cancer.

## 4. Discussion

This study provides an integrative transcriptomic framework describing the transition from circulating tumor cell–negative (CTC^−^) to circulating tumor cell–positive (CTC^+^) states in breast cancer. Through systematic bioinformatics analyses, spanning differential expression, enrichment mapping, network modeling, survival analysis, and drug perturbation screening, we reveal that CTC^+^ cells exhibit extensive immune suppression, chromatin remodeling, and signaling activation, whereas CTC^−^ patients already display transcriptional alterations indicative of systemic pre-metastatic remodeling. Together, these findings support a working continuum model of CTC-associated molecular remodeling, in which the observed CTC^−^ and CTC^+^ transcriptional states may represent biologically related stages of increasing metastatic adaptation.

### 4.1. CTC^+^ Cells Exhibit Coordinated Immune Evasion and Epigenetic Activation

Our data demonstrates that CTC^+^ samples are characterized by widespread repression of immune signaling pathways, including complement activation, antigen presentation, and adaptive immune responses. This transcriptional silencing mirrors previous observations that CTCs adopt “immune-quiet” phenotypes to evade immune surveillance during circulation [31].

Downregulation of *CD79A*, *PPP2R2B*, and *IGHG* family members reflects diminished B-cell receptor (BCR) and antibody-mediated activity, suggesting a strategy of immune invisibility akin to immunoediting processes described by Schreiber et al. [32]. Simultaneously, upregulation of *HDAC1* and *JUN* indicates a shift toward epigenetic and transcriptional plasticity, enabling CTCs to adapt to dynamic microenvironments. HDAC-mediated chromatin remodeling has been shown to facilitate epithelial–mesenchymal transition (EMT) and metastatic dissemination in breast cancer [33].

### 4.2. Primary Tumors from CTC^−^ Patients Display Early Systemic Reprogramming Before Detectable Dissemination

A striking observation of this study is the presence of extensive transcriptional changes in primary tumors from CTC^−^ patients compared to normal breast tissue controls. Despite the absence of detectable CTCs, over 3400 genes were significantly dysregulated, implicating pathways such as MAPK/ERK signaling, receptor tyrosine kinase (RTK) activation, and immune modulation. These alterations may reflect tumor-intrinsic and microenvironmental programs associated with the CTC^−^ state, potentially mediated by soluble factors, exosomes, or early immune crosstalk [34].

In CTC^−^ state, the downregulation of *PDHA1*, *NFIB*, and *CCDC8* suggests alterations in stress-response and metabolic pathways, consistent with pre-metastatic niche formation. Similarly, upregulated genes (*RTKN*, *ACTC1*, *FAM110A*) point to cytoskeletal and signaling activation prior to CTC emergence.

Together, these data suggest that molecular remodeling occurs before tumor cells enter the circulation, supporting the concept of a “molecularly primed” bloodstream environment, as previously proposed in early metastasis models [35]. However, because the analysis is cross-sectional, these differences cannot be interpreted as direct evidence that such alterations temporally precede CTC emergence.

### 4.3. Prognostic Relevance of CTC-Associated Gene Signatures

The identified gene panels derived from CTC^+^ and CTC^−^ comparisons were able to stratify patient survival in the TCGA-BRCA cohort with high significance. For instance, the *KLK10*/*MEOX2*/*IRS4*/*RND2* signature yielded a hazard ratio (HR) of 1.74 (*p* = 0.00215), while the *PPP2R2B*/*IGF1R*/*CD79A* cluster achieved an HR of 2.44 (*p* < 10^−6^). These findings support the potential prognostic relevance of multigene transcriptional modules rather than single-gene biomarkers, aligning with modern network-based prognostic frameworks [36], although independent clinical validation is required before clinical utility can be established.

Notably, *ALDH3A1* emerged as a potentially novel tumor suppressor, with lower expression correlating with poorer survival outcomes (HR = 6.51). Its known role in oxidative stress detoxification suggests that CTCs may deliberately suppress ALDH3A1 to tolerate oxidative stress during bloodstream transit. This finding introduces *ALDH3A1* as a candidate CTC-specific prognostic biomarker deserving experimental validation.

### 4.4. Therapeutic Implications: Drug Repurposing Opportunities

Drug perturbation analysis using the L1000CDS^2^ platform revealed several compounds predicted to reverse the CTC^+^ transcriptional signature, including Trichostatin A (HDAC inhibitor), BX-795 (TBK1/IKKε inhibitor), and Alvocidib (CDK inhibitor). The recurrent identification of HDAC inhibitors aligns with our finding of *HDAC1* upregulation, underscoring the potential for epigenetic reprogramming therapies to target CTC persistence.

Similarly, kinase inhibitors such as Foretinib and PD-184352, identified in the CTC^−^ context, target MAPK and RTK signaling pathways that were enriched in this group. These compounds could thus serve as candidate preventive interventions in early-stage disease by suppressing the transcriptional priming of potential CTC precursors.

### 4.5. A Transcriptional Continuum from CTC^−^ to CTC^+^ States

Integrating all findings, we propose a three-stage working model of CTC-associated breast cancer biology. Importantly, this model represents a conceptual interpretation of the cross-sectional molecular patterns observed in the present study and does not imply a directly demonstrated temporal sequence:

1. Pre-CTC (CTC^−^) stage: Systemic immune and metabolic reprogramming occurs in the host’s blood cells prior to CTC release.
2. CTC^+^ stage: Tumor cells entering the circulation activate epigenetic and survival programs while repressing immune visibility.
3. Metastatic stage: These transcriptional traits converge with metastatic gene networks observed in advanced breast cancer.

This model aligns with the continuum hypothesis that metastasis arises through gradual adaptation rather than binary transition. We further hypothesize that targeting the transcriptional intermediates of this continuum, before full metastatic commitment, may represent a viable therapeutic strategy. However, longitudinal studies are required to determine whether individual tumors progress through these states in a temporal sequence.

### 4.6. Limitations and Future Directions

While the study leverages an integrative and robust bioinformatics framework and survival analysis of CTC-associated transcriptional states, several limitations should be considered. First, the relatively small cohort size (CTC^+^, *n* = 8; CTC^−^, *n* = 9; normal controls, *n* = 5) limits statistical power and the generalizability. Second, the study represents a secondary analysis of a publicly available dataset and is therefore dependent on the clinical and technical metadata available from the source study. Potential differences in clinicopathological characteristics between the CTC^+^ and CTC^−^ groups, including molecular subtype, disease stage, age, and treatment status, may also contribute to the observed transcriptional differences and represent potential sources of confounding. Given the limited sample size, robust subtype-stratified or multi-variable analyses were not feasible, and the findings should therefore be regarded as exploratory and hypothesis-generating. Third, the cross-sectional design does not allow direct demonstration of a temporal transition from CTC^−^ to CTC^+^ status or establishment of causal relationships between the identified molecular programs and CTC emergence.

Importantly, the RNA-seq profiles analyzed in this study were derived from bulk primary breast tissues rather than isolated CTCs. Consequently, the observed signatures should be interpreted as primary-tumor transcriptional programs associated with CTC status and may reflect contributions from tumor cells as well as stromal and immune components. Experimental validation is therefore required to establish the functional relevance of the identified hub genes, pathways, and predicted drug candidates. Future work should focus on validating key hub genes (*HDAC1*, *ALDH3A1*, *PPP2R2B*) and pathway-level findings in independent clinical samples and appropriate experimental models, investigating the mechanisms underlying immune-evasion networks, and pharmacologically testing the top-ranked candidate compounds. Future studies should combine larger, prospectively characterized cohorts with matched primary-tumor and CTC profiling, functional experimental models, and single-cell or spatial transcriptomic approaches to resolve cell-type-specific mechanisms underlying CTC-associated disease biology.

## 5. Conclusions

This integrative transcriptomic study delineates a dynamic molecular continuum between CTC-negative (CTC^−^) and CTC-positive (CTC^+^) breast cancer patients, revealing how tumor cells progressively acquire the molecular traits necessary for hematogenous dissemination and immune evasion. Our results demonstrate that

- CTC^+^ samples exhibit marked transcriptional reprogramming characterized by immune suppression, epigenetic activation, and cytoskeletal remodeling, consistent with a metastasis-competent phenotype.
- CTC^−^ patients, despite lacking detectable circulating cells, show significant systemic molecular alterations involving MAPK/ERK and RTK signaling, suggesting a pre-metastatic reprogramming state.
- Multi-gene prognostic models derived from CTC-associated hub genes (*ALDH3A1*, *PPP2R2B*, *KLK10*, *MEOX2*, *IRS4*) robustly stratified patient survival, underscoring their potential as liquid-biopsy–based biomarkers.
- Drug repurposing analysis identified HDAC inhibitors (Trichostatin A) and CDK inhibitors (Alvocidib) as promising candidates capable of reversing the CTC^+^ expression profile.
- Together, these findings suggest that metastasis evolves not as a discrete event but through gradual transcriptional adaptation, from immune-modulated CTC^−^ states to fully adaptive CTC^+^ phenotypes. Targeting these transcriptional intermediates could enable early interception of metastasis, marking a shift toward precision medicine strategies that exploit the molecular vulnerabilities of circulating tumor cells.

## Figures and Tables

**Table 1:** Clinicopathological and molecular characteristics of the breast cancer samples according to CTC status.

Characteristic	CTC^+^ (*n* = 8)	CTC^−^ (*n* = 9)	Normal Controls (*n* = 5)
Hormone receptor positive, n (%)	7 (87.5)	6 (66.7)	N/A
HER2 positive, n (%)	1 (12.5)	3 (33.3)	N/A
Luminal A, n (%)	4 (50.0)	2 (22.2)	N/A
Luminal B, n (%)	2 (25.0)	3 (33.3)	N/A
HER2-enriched, n (%)	1 (12.5)	3 (33.3)	N/A
Triple-negative breast cancer, n (%)	1 (12.5)	1 (11.1)	N/A

**Table 2:** BRCA cell lines sensitive to compounds with potential to reverse CTC^+^ signature gene expression.

Compound	Cell Line	IC50 (μM)	Compound	Cell Line	IC50 (μM)
Alvocidib (Flavopiridol)	AU565	0.12	BX-795 ^1^	BT-549	6.63
	COLO-824	0.14		CAL-120	4.95
	DU-4475	0.09		CAL-51	1.33
	HCC1187	0.07		DU-4475	1.76
	HCC1599	0.06		EVSA-T	3.43
	HCC2157	0.16		HCC1187	3.13
	HCC70	0.10		Hs-578-T	3.24
	MCF7	0.11		JIMT-1	6.23
	MDA-MB-157	0.20		MDA-MB-157	1.56
	MDA-MB-468	0.14		MDA-MB-175-VII	4.95
	MFM-223	0.18		MDA-MB-231	5.96
	MRK-nu-1	0.12		MDA-MB-468	1.59
	OCUB-M	0.16		OCUB-M	6.17
Trichostatin A	MCF7	0.02	BX-795 ^2^	HCC1569	6.91

^1^ GDSC1; ^2^ GDCS2.

## Data Availability

The dataset analyzed during the current study are available at NCBI/SRA database with project no: PRJNA839244: https://www.ncbi.nlm.nih.gov/sra/?term=PRJNA839244 (accessed on 9 August 2025).

## Abbreviations

The following abbreviations are used in this manuscript:

BCR	B-cell receptor
BRCA	Breast invasive carcinoma
CAM	Cell adhesion molecule
C-indices	Concordance indices
CTC	Circulating tumor cell
CTC^+^	Circulating tumor cell-positive
CTC^−^	Circulating tumor cell-negative
DEG	Differentially expressed gene
ECM	Extracellular matrix
EMT	Epithelial–mesenchymal transition
GDSC	Genomics of Drug Sensitivity in Cancer
HR	Hazard ratio
KEGG	Kyoto Encyclopedia of Genes and Genomes
PPI	Protein–protein interaction
RNA-seq	RNA sequencing
RTK	Receptor tyrosine kinase
SRA	Sequence Read Archive
t-SEN	t-distributed stochastic neighbor embedding
TCR	T-cell receptor
VST	Variance stabilizing transformation
